# Energy Consumption Model of SCHC Packet Fragmentation over Sigfox LPWAN

**DOI:** 10.3390/s22062120

**Published:** 2022-03-09

**Authors:** Sergio Aguilar, Antonis Platis, Rafael Vidal, Carles Gomez

**Affiliations:** Department of Network Engineering, Universitat Politècnica de Catalunya, 08860 Castelldefels, Spain; antonios.platis@estudiantat.upc.edu (A.P.); rafael.vidal@entel.upc.edu (R.V.); carlesgo@entel.upc.edu (C.G.)

**Keywords:** IoT, LPWAN, SCHC, Sigfox, fragmentation, IETF, ACK-on-Error, energy model

## Abstract

The Internet Engineering Task Force (IETF) has standardized a new framework, called Static Context Header Compression and fragmentation (SCHC), which offers adaptation layer functionality designed to support IPv6 over Low Power Wide Area Networks (LPWANs). The IETF is currently profiling SCHC, and in particular its packet fragmentation and reassembly functionality, for its optimal use over certain LPWAN technologies. Considering the energy constraints of LPWAN devices, it is crucial to determine the energy performance of SCHC packet transfer. In this paper, we present a current and energy consumption model of SCHC packet transfer over Sigfox, a flagship LPWAN technology. The model, which is based on real hardware measurements, allows to determine the impact of several parameters and fragment transmission strategies on the energy performance of SCHC packet transfer over Sigfox. Among other results, we have found that the lifetime of a device powered by a 2000 mAh battery, transmitting packets every 5 days, is 168 days for 2250-byte packets, while it increases to 1464 days for 77-byte packets.

## 1. Introduction

In the last years, Low Power Wide Area Network (LPWAN) technologies have gained significant momentum as solutions for providing connectivity to Internet of Things (IoT) devices. LPWANs typically offer unique characteristics, such as a simple star network topology, a link range in the order of several kilometers, and low energy consumption, which enables multiyear lifetimes for battery-operated IoT devices [1]. Relevant LPWAN technologies include Sigfox, LoRaWAN, NB-IoT, and IEEE 802.15.4 w [2].

LPWAN technologies typically support low bit rates, which contribute to achieving long link range. However, packet transmission at a low bit rate leads to high energy consumption due to a high packet transmission time. In order to limit the impact of this problem, many LPWAN technologies are characterized by a short Layer 2 (L2) frame size. On the other hand, LPWAN devices can benefit from Internet connectivity by supporting IPv6. However, IPv6 requires its underlying layer to support a Maximum Transmission Unit (MTU) of at least 1280 bytes [3]. If an LPWAN technology does not offer packet Fragmentation and Reassembly (F/R) functionality, such as Sigfox or LoRaWAN, an adaptation layer including F/R support is needed between IPv6 and the underlying LPWAN technology. To this end, the IETF has recently developed a standard called Static Context Header Compression and fragmentation (SCHC) [4].

SCHC provides adaptation layer functionality that includes F/R. SCHC F/R allows to transmit packets even if they have a size greater than the supported L2 MTU. SCHC has been purposefully specified following a generic approach, since it was envisioned that SCHC would be used on top of several technologies. For each LPWAN technology, a companion specification called a SCHC Profile determines which SCHC components need to be supported and how they are configured [5,6,7].

The SCHC F/R process is performed at the expense of contributing header and message overhead to packet transmission. Considering the energy constraints of IoT devices (many of which are not mains-powered), it is fundamental to evaluate the energy performance of SCHC fragmented packet transmission. However, to the best of our knowledge, there is no previous work in the literature on this topic [8,9,10,11,12,13,14,15,16].

In this paper, we model and evaluate the energy performance of SCHC packet fragmentation over Sigfox, a flagship LPWAN technology that supports a severely constrained maximum payload size (i.e., 12 bytes) for IoT device packet transmission. Among others, our results quantify how the lifetime of a battery-operated device performing periodic packet transfers over Sigfox increases with the period between transfers and decreases with packet size. For example, assuming a battery capacity of 2000 mAh, and a period of 5 days, the device lifetime increases from 168 days (for a 2250-byte packet) to 1464 days (for a 77-byte packet). We also evaluate the impact on performance of different fragment transmission strategies, as well as device hardware features.

The rest of the paper is organized as follows: Section 2 presents related work. Section 3 overviews the main features of Sigfox. Section 4 describes the main features of SCHC F/R and how it is supported over Sigfox. Section 5 provides our current consumption model of SCHC packet fragmentation over Sigfox. In Section 6, the model is used to evaluate the energy performance of SCHC packet fragmentation over Sigfox. Section 7 concludes the paper.

## 2. Related Work

This section reviews related work. First, we focus on literature regarding the energy performance of Sigfox. Secondly, we overview evaluation studies on SCHC F/R.

### 2.1. Sigfox Energy Performance

The energy performance of the Sigfox technology has been a topic of interest for the research community. Martinez et al. [17] provided a general energy consumption model for IoT devices, including Sigfox devices, but did not provide information regarding the considered Sigfox module, nor device lifetime. In [18], authors provided an analytical model that characterizes Sigfox in terms of device current consumption, device lifetime, and energy cost of data delivery. Their results show that, using a MKRFOX1200 development kit with an ATA8520 Sigfox module, with a battery of 2400 mAh, a theoretical device lifetime of 1.5 years is possible when sending one message every 10 min at 100 bit/s. In [19], authors evaluated theoretically the energy consumption of different LPWANs for over-the-air updates. Results show that full firmware updates consume a significant amount of energy, especially for low bit rate technologies such as Sigfox. The datasheet values of an Onsemi AXSF Sigfox module were considered in their evaluation.

Hernandez et. al. [20] performed extensive energy consumption measurements; their results show that IoT sensors using Sigfox technology can be autonomous during remarkably long periods of time, with a lifetime of up to 4 years, when sending a message every 60 min at 100 bit/s and operating on a 1000 mAh battery. The evaluation was based on a Telit LE51-868/DIP Sigfox module. Morin et. al. [21] compared different wireless technologies, such as Sigfox, LoRaWAN, and Bluetooth Low Energy (BLE) in terms of device lifetime. It was found that a Sigfox device, running on two AAA batteries (of 1250 mAh at 1.5 V each) can achieve a lifetime of 25 years when sending 10 bytes per day at 100 bit/s. Ogawa et al. [22] estimated the energy cost of Sigfox transmissions, using the TD1207R/08R module.

An IoT solution for art conservation using Sigfox was presented in [23]. That work shows that if a device with a 1700 mAh battery is sending one sample per hour, it is possible to achieve a lifetime of 1.5 years, using the Telit LE51-868S Sigfox module. Moreover, authors indicate that aggregation strategies (i.e., sending more than one sample per transmission) can improve energy consumption and extend the node lifetime to 5 years. Authors in [24] concluded that, in cases where extremely long range is required, Sigfox has better device battery lifetime for small daily throughputs than other LPWAN technologies. Their theoretical evaluation was based on the AX-Sigfox module. Similar results are presented in [25]. In [26], Lykov et al. studied Sigfox, using the AX-SIP-SFEU radio module. The authors found that, for a battery capacity of 2000 mAh, a payload size increase will reduce the device lifetime by up to 18 days, and that a daily message rate increase (up to 140 messages/day) can reduce the device lifetime down to 209 days.

Table 1 presents a summary of published works that evaluate Sigfox energy performance. None of these studies modeled the energy consumption of packet fragmentation over Sigfox.

### 2.2. SCHC F/R

Authors in [8] showed that SCHC packet fragmentation can increase reliability, with a trade-off in terms of energy consumption and goodput. The same authors analyzed in [9] the use of SCHC packet fragmentation to reduce network congestion and increase network capacity. An overview and a simple evaluation showing the header and message overhead of SCHC F/R is presented in [2]. Other performance metrics, such as total channel occupancy, goodput and total delay were studied in [10], over an ideal channel. Optimal configuration values for SCHC F/R over LoRaWAN and Sigfox were provided in [11]. SCHC Receiver-Feedback Techniques (RFTs) and alternative RFTs were presented and evaluated in [12] over LoRaWAN, as part of a reliable fragmentation method. Results show that alternative RFTs may be optimal depending on the error rate and pattern, providing greater efficiency.

Since the publication of the base SCHC specification [4], the IETF LPWAN WG has been developing SCHC Profiles, which provide configurations of SCHC F/R functionality tailored to specific LPWAN technologies, such as Sigfox [6], LoRaWAN [5], and NB-IoT [7]. Sanchez-Gomez et al. [13] presented an evaluation of the LoRaWAN SCHC Profile in a real testbed. SCHC F/R provided benefits in terms packet delivery ratio, with a processing time overhead below 8 ms and a memory usage of only 609 bytes. Santa et al. [14] used SCHC to support IPv6 over LoRaWAN and NB-IoT for personal mobility vehicles. NB-IoT showed lower latency and low fragment error rate; however, it consumed more power than LoRaWAN. In [15], SCHC fragmentation over Sigfox was overviewed and evaluated theoretically and empirically by using a LoPy4 module, in terms of transfer time and number of Sigfox uplink and downlink messages. Muñoz et al. [16] evaluated SCHC over LoRaWAN and obtained a model to determine channel occupancy efficiency based on LoRaWAN and SCHC configuration parameters.

Table 2 summarizes the works related to SCHC F/R performance evaluation, along with the performance parameters and the method used. Together, these studies provide important insights into SCHC F/R. However, they neither provide a detailed model of, nor evaluate, the current consumption or the energy performance of SCHC Packet transfer.

To the best of our knowledge, no previous work provides a current or energy consumption model nor evaluates the energy performance of fragmented packet transfer with SCHC.

## 3. Sigfox

This section overviews the Sigfox technology. The section is organized into four subsections that focus on the following features of Sigfox, respectively: network architecture, physical layer features, communication procedures, and frame formats.

### 3.1. Network Architecture

**Figure 1:**
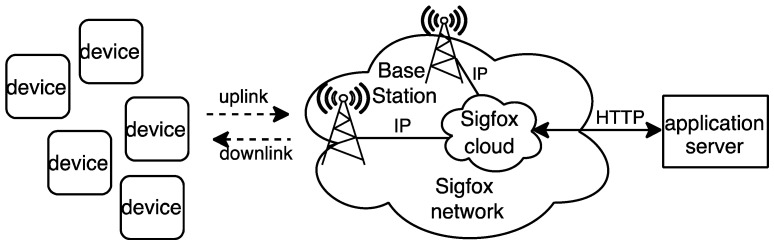
Sigfox network architecture.

Figure 1 shows the main elements of the Sigfox network architecture. Sigfox is based on a star of stars topology composed of three main parts: the device (i.e., the IoT device such as a sensor or an actuator), the Sigfox Network (which comprises Base Stations and the Sigfox cloud), and the application server (where the end-user application is hosted). The device sends data that may be received by one or more Base Stations, which provides spatial diversity. Base Stations send the messages received to the Sigfox cloud over IP. The latter is in charge of message reconstruction and message duplication avoidance. Messages are then forwarded from the Sigfox cloud to the application server by using HTTP (e.g., an HTTP POST message with a JSON payload). The application server may respond after the receipt of a message with data to be sent to the device.

### 3.2. Physical Layer Features

Sigfox is a global network operator for IoT devices that uses unlicensed sub-GHz frequency bands, ranging from 862 MHz to 928 MHz. Due to local spectrum access regulations in different countries and world regions, Sigfox defines seven geographical zones, each one with a specific Radio Configuration (RC), called RC1 to RC7, respectively. An RC establishes a particular set of operation frequencies, output transmit power, spectrum access mechanism and Uplink (UL) bitrate. The UL bitrate may either be 100 bit/s or 600 bit/s, while the Downlink (DL) bitrate is 600 bits/s in all RCs.

In RC1 (which is defined for Europe, Middle East, and Africa), the operational frequencies are 868.130 MHz and 869.525 MHz for the UL and the DL, respectively. The system supports using different frequency channels over one 192 kHz band for UL transmission and over another 192 kHz band for DL transmission. Sigfox uses Ultra Narrow Band (UNB) transmission, which is intended to overcome noise and interference issues. The channel bandwidth is 100 Hz and 1.5 kHz for UL and DL channels, respectively. DBPSK and GPSK modulations are used for UL and DL transmission, respectively, for the sake of spectrum efficiency, base station sensitivity, and device cost.

In order to comply with spectrum access regulations, RC1 defines a duty cycle limitation of 1% and 10% in the UL and in the DL, respectively, that must be enforced in a per-hour basis. To this end, Sigfox allows a device in RC1 to send only up to 6 full-sized UL messages per hour.

### 3.3. Communication Procedures

Sigfox defines two communication procedures: the Uplink Procedure (U-Proc), which is used by the device to transmit data messages to the network, and the Bidirectional Procedure (B-Proc), which allows the device to transmit an UL message and enable an opportunity for a subsequent DL message. We next describe both communication procedures.

#### 3.3.1. U-Proc

A U-Proc is carried out by a device in order to send an UL frame. The device can start a U-Proc at any moment, provided that spectrum access regulations of the corresponding RC are followed.

**Figure 2:**
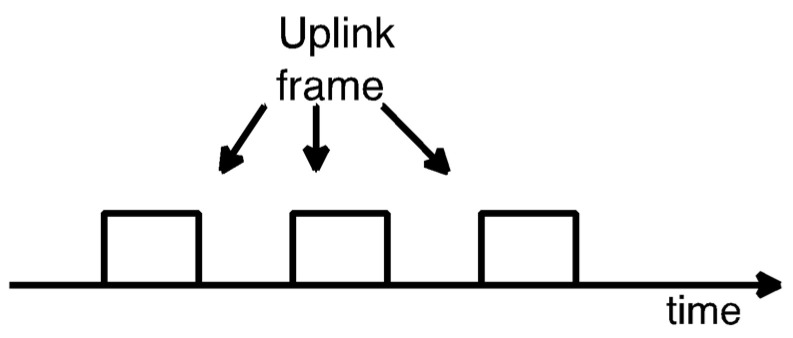
U-Proc example. An UL frame is transmitted three times, using a different frequency channel in each case.

Sigfox UL transmission supports time and frequency diversity. The UL frame is transmitted three times, where each one of the three transmissions is performed over a different frequency channel. Figure 2 shows an example of a U-Proc.

#### 3.3.2. B-Proc

**Figure 3:**
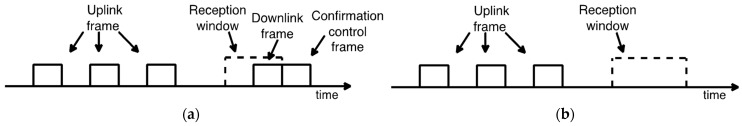
B-Proc examples: (**a**) a DL frame is received, and a confirmation control frame is sent; (**b**) no DL frame is received by the device.

In Sigfox, DL transmission requires the device to perform a B-Proc, whereby the device first transmits an UL frame. The UL frame header carries a field that indicates that the device will allow a response from the network. The device opens a reception window, and the network or the application server may choose to send data, if available, or wait for another DL opportunity. If no message is received by the device, the reception window will be closed after *T_RX MAX_* (i.e., 25 s in RC1) time has passed. If a DL frame is received by the device, the latter transmits an UL confirmation control frame. Figure 3a,b illustrates the B-Proc when a DL frame is received and when it is not received, respectively.

### 3.4. Frame Formats

This section briefly describes the data frame formats supported in Sigfox, for both the UL and the DL.

The UL frame is composed of a UL frame payload, with a size that may vary from 0 to 96 bits. Additionally, the Sigfox protocol prepends an 81-bit header and appends between 32-bit to 56-bit tail bits to the UL frame payload. The Sigfox protocol header fields include a Preamble, a Frame Type, a Length Indicator, a Bidirectional Flag, a Repeated Flag, a Message Counter, and a Device ID. The Bidirectional Flag indicates if the UL frame corresponds to a U-Proc or a B-Proc. The tail includes Message Authentication and Cyclic Redundancy Check (CRC) fields.

The confirmation control frame shares the same format as the UL frame and carries information of the status of the MCU, such as the battery voltage value during and in the absence of radio transmissions, the MCU temperature and the signal strength estimated while receiving the DL frame.

The DL frame contains a fixed size 64-bit DL frame payload; therefore, padding bits must be added if a shorter data payload size needs to be transferred. A Sigfox protocol header of 136 bits is prepended to the DL frame payload, including a Preamble, a Field type, and an Error Correction Code (ECC). In a similar way to the UL frame, the tail of the DL frame includes Message Authentication and CRC fields. This tail has a size of 24 bits.

## 4. SCHC over Sigfox

In this section, we introduce the basic concepts of the SCHC over Sigfox Profile (in short, SCHC over Sigfox), focusing on how SCHC F/R is used over Sigfox radio links to enable reliable fragmented packet transfer.

The current version of SCHC over Sigfox [6] includes a reliable F/R mode called ACK-on-Error. This mode supports selective fragment retransmission along with receiver feedback given by messages called SCHC ACKs, which report whether SCHC Fragments have been successfully received or not.

The next subsections describe the main tools used to support reliable packet fragmentation by means of ACK-on-Error, the functionality provided by this mode, and how it is configured when used over Sigfox.

### 4.1. Tiles, Windows and Bitmaps

**Figure 4:**
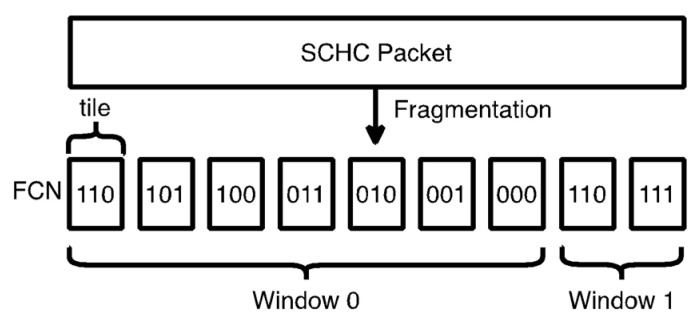
SCHC Fragmentation process. The SCHC Packet is fragmented into tiles, which are numbered and grouped into windows. In this example, WINDOW_SIZE is equal to 7, and the FCN field has a size of 3 bits. The last FCN value, which corresponds to the last tile, has all bits set to 1 (i.e., the FCN is 7).

In SCHC, the data unit to be transferred is called a SCHC Packet. If the SCHC Packet is larger than the L2 maximum payload size (e.g., 12 bytes), it is fragmented in smaller units called tiles (see Figure 4). In ACK-on-Error, tiles have a fixed size, except for the last one, which can be smaller (see Section 4.2 for further details). Tiles are carried as the payload of data units called SCHC Fragments. Tiles are numbered using an identifier called Fragment Compressed Number (FCN). A special FCN value with all bits set to 1 indicates the last tile of the SCHC Packet.

A group of WINDOW_SIZE tiles is called a window (see Figure 4). Each window required to transfer a SCHC Packet is sequentially numbered from 0 upwards. Tiles are numbered per window, sequentially, from WINDOW_SIZE-1 downwards. A specific combination of window number and FCN uniquely identifies each tile.

A SCHC ACK provides feedback to a fragment sender by means of an encapsulated bitmap. A bitmap is a sequence of bits, where each bit indicates whether a specific SCHC Fragment from a given window has been successfully received or not. The size of a bitmap is equal to WINDOW_SIZE. Bitmap bits are ordered from the most significant bit, corresponding to the tile with number WINDOW_SIZE-1, to the least significant bit, corresponding to tile 0 (for all windows except the last one) or the last fragment of the whole fragmented SCHC Packet (for the last window). If a bitmap bit is set to 1, it indicates successful reception of the corresponding SCHC Fragment, whereas value 0 indicates otherwise.

### 4.2. SCHC Messages

**Figure 5:**
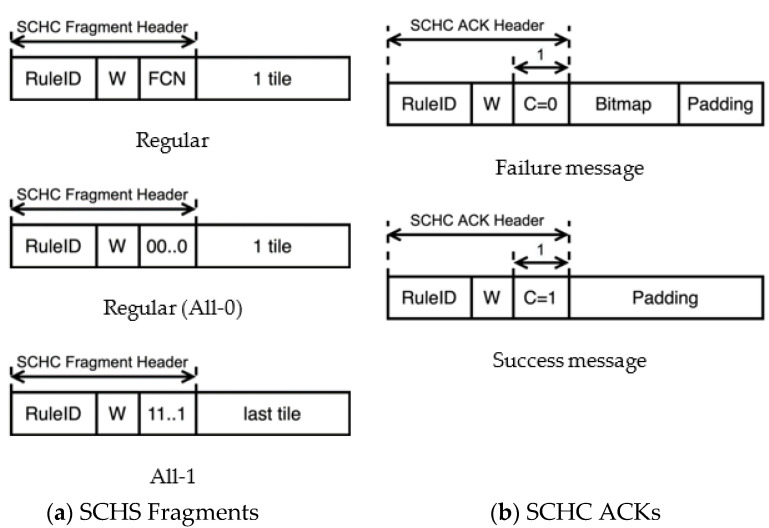
SCHC Message formats for SCHC over Sigfox: (**a**) Regular SCHC Fragment, Regular (All-0) SCHC Fragment (FCN = 00..0), and All-1 SCHC Fragment (FCN = 11..1); (**b**) SCHC ACK failure message (C = 0) and SCHC ACK success message (C = 1).

The SCHC over Sigfox specification adapts the message formats presented in the generic SCHC specification, which includes SCHC Fragments and SCHC ACK messages. The formats of these SCHC messages are shown in Figure 5.

A SCHC Fragment is composed of a SCHC Fragment Header and a payload. In SCHC over Sigfox, the payload carries exactly one tile, and the SCHC Fragment Header contains the window number (W) and the FCN for the tile carried by the SCHC Fragment. These fields are preceded by another field called the RuleID, which indicates the size of the SCHC Fragment Header for a given SCHC Packet transfer. There are two kinds of SCHC Fragments: (i) the Regular SCHC Fragments and (ii) the All-1 SCHC Fragments. Regular SCHC Fragments are used to carry any tile of a window, except for the last tile of the last window. The last tile of each window (except for the last window) is numbered with an FCN of 0, and it is carried by a so-called All-0 SCHC Fragment. The All-1 SCHC Fragment carries the last tile of the last window and signals the end of the SCHC Packet.

The SCHC ACK message format has two parts: the SCHC ACK Header and the bitmap. The SCHC ACK Header comprises three fields: the RuleID, the window number (W), and an integrity check bit (C). The RuleID carries the same value as the SCHC Fragments for which receiver feedback is provided. The W field indicates the window number the SCHC ACK corresponds to. The C bit reports on the status of the received SCHC Packet. C = 1 indicates a successful SCHC Packet transfer, whereas C = 0 indicates otherwise. As an optimization, when C = 1, a bitmap is not included. If needed, padding is added to the SCHC ACK to align its size with the minimum data unit supported by the underlying L2.

**Figure 6:**
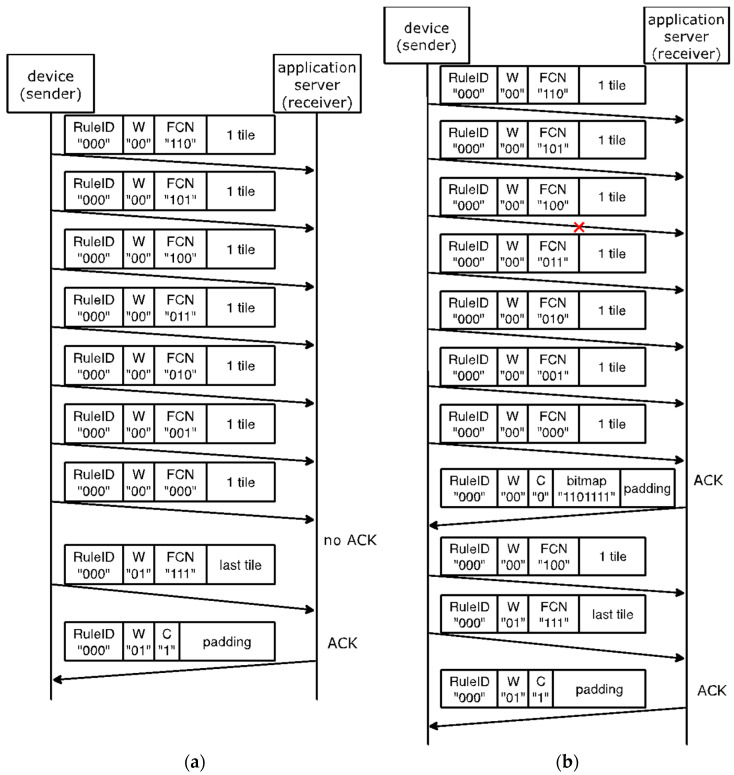
Examples of an 88-byte SCHC Packet transfer over Sigfox. The SCHC Packet is carried by 8 SCHC Fragments and 2 windows (with numbers 00 and 01). Since the SCHC Packet size is not greater than 300 bytes, a single-byte SCHC Header is used: (**a**) no SCHC Fragment losses; (**b**) one SCHC Fragment loss.

Figure 6 shows two SCHC Packet transfer examples, with and without SCHC Fragment losses. After receiving an All-0 SCHC Fragment, for the sake of efficiency, a SCHC ACK is only sent by the receiver if any SCHC Fragment from the corresponding window has been lost (see Figure 6b). A SCHC ACK is sent unconditionally at the end of the whole fragmented SCHC Packet transfer (i.e., after the All-1 SCHC Fragment).

### 4.3. ACK-on-Error Configuration

To reliably transfer a SCHC Packet of a size up to 300 bytes, SCHC over Sigfox recommends the use of a single-byte SCHC Header. In that case, the SCHC Fragment header is composed of a 3-bit RuleID, a 2-bit W, a 3-bit FCN, and a fixed tile size (t) of 11 bytes. WINDOW_SIZE is 7.

For SCHC Packet sizes greater than 300 bytes, and up to 2250 bytes, SCHC over Sigfox recommends using a two-byte SCHC Header. In this case, the 16 SCHC Fragment header bits are distributed as follows: an 8-bit RuleID, a 3-bit W, a 5-bit FCN, and t equal to 10 bytes. In this case, WINDOW_SIZE is 31.

## 5. Modeling SCHC F/R over Sigfox Current Consumption

In this section, we present models of crucial energy performance parameters of SCHC F/R over Sigfox, such as device current consumption, device lifetime and energy cost. For the models, we assume a Sigfox device that sends SCHC Packets to the Sigfox network. Such behavior may correspond to an IoT device sending sensor readings.

We first introduce the experimental setup used to perform current consumption measurements on a real device. Second, we identify the different states of a device that performs reliable SCHC Packet transfer by using ACK-on-Error over Sigfox, and we obtain their corresponding current and energy consumption profiles. Finally, we model the current and energy consumption of fragmented SCHC Packet transfers, considering single and periodic transfers. For the latter, we also model the lifetime of a battery-operated device.

### 5.1. Experimental Setup

**Figure 7:**
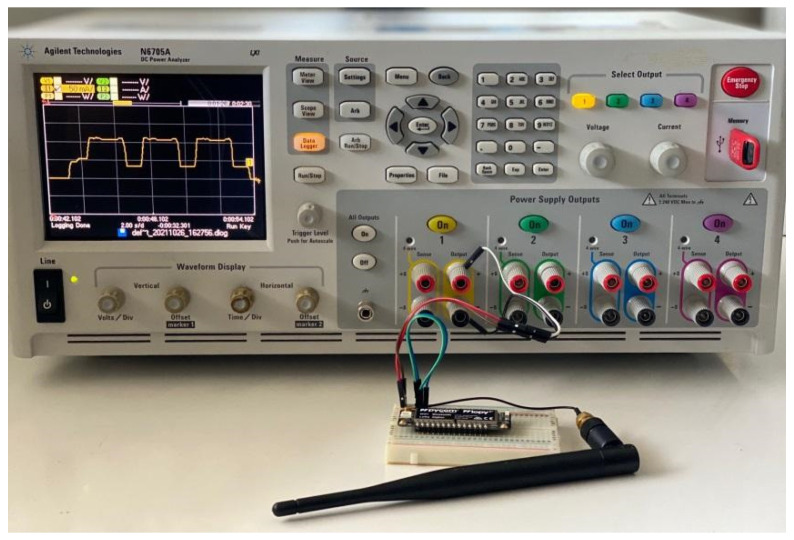
Experimental setup with the Sigfox device and the power analyzer used.

Our models are derived from current consumption measurements on a real Sigfox device: a Pycom LoPy4 development board [27]. Figure 7 shows the experimental setup, which includes an Agilent N6750A power analyzer and the Sigfox device. The experiments were carried out in an indoor environment in the city of Castelldefels, in Spain. The Sigfox coverage in the scenario is near-ideal, with negligible frame loss rate.

The LoPy4 module is based on the Espresiff ESP32 MCU. The latter includes a Wi-Fi and a Bluetooth interface, along with a Sigfox Semtech SX1276 radio module. Note that the LoPy4 module also has a built-in RGB LED. In our measurements, the LoPy4 board was programmed to enable the Sigfox radio interface and shut down other radio modules and peripherals (including the Wi-Fi and BLE interfaces and the RGB LED) on boot.

The LoPy4 has a voltage regulator, which supports input voltages between 3.5 V and 5.5 V. The output voltage of the regulator is 3.3 V. In all measurements performed, the supplied voltage is 3.5 V.

The Sigfox radio of the LoPy4 board is configured for RC1. Accordingly, the UL data rate is 100 bps, and the DL data rate is 600 bps. The transmit power is +14 dBm. The receiver sensitivity is −126 dBm.

The SCHC over Sigfox implementation used in our evaluation is based on the one presented in [15], which is publicly available.

### 5.2. SCHC Packet Transfer States

In order to comply with the duty cycle constraints in RC1, SCHC Fragments may be sent by using different approaches. In our model, we consider two possible options: (i) sending one SCHC Fragment per cycle of 10 min (and sleeping otherwise), and (ii) sending up to 6 SCHC Fragments back to back per cycle of 60 min (and sleeping otherwise).

Let *N_pC_* denote the number of SCHC Fragments that are sent back-to-back per cycle, where $1\leq N_{pC}\leq 6$. Let *N_C_* denote the number of cycles required to complete a SCHC Packet transfer.

**Figure 8:**
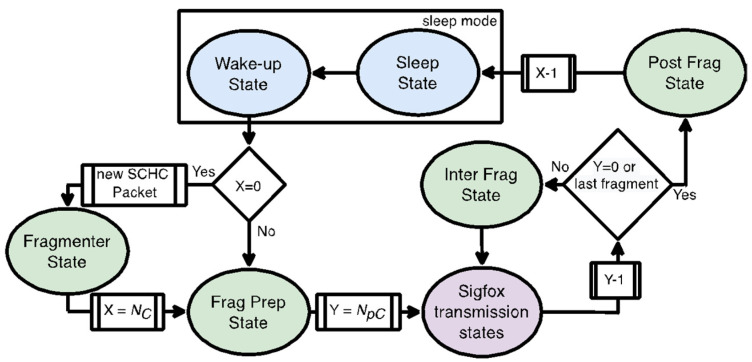
Fragmented SCHC Packet transfer state diagram. Sleep and Wake-up states, SCHC Fragmentation-related states, and Sigfox transmission states are depicted in blue, green, and purple, respectively. X and Y variables correspond to the number of cycles (*Nc*) and to the number of SCHC Fragments per cycle (*N_pC_*), respectively.

Each cycle comprises several states (see Figure 8). Initially, the device is sleeping (Sleep state), and then, the device wakes up (Wake-up state). If a new SCHC Packet needs to be sent, the device enters the Fragmenter state, where SCHC Packet fragmentation is performed. In this state, the device creates the SCHC Fragments from the SCHC Packet, which includes selecting the appropriate RuleID (according to the SCHC Packet size) and the corresponding FCN and W values for each SCHC Fragment.

After the Fragmenter state or after the Wake-up state if the device continues sending an already fragmented SCHC Packet, the device reaches the Frag Prep state, where it prepares the next SCHC Fragment to be sent and selects the Sigfox transmission procedure to be used for this SCHC Fragment. The prepared SCHC Fragment is then sent accordingly (the device is in the Sigfox transmission state). When more than one SCHC Fragment is sent per cycle $N_{pC}\geq 2)$, the device enters the Inter Frag state to prepare the next SCHC Fragment to be transmitted or to process a SCHC ACK (when available).

Finally, after sending all the SCHC Fragments of a cycle, or after sending the last SCHC Fragment of a SCHC Packet, the device reaches the Post Frag state. In this state, the device processes a SCHC ACK (when available) and returns to the Sleep State. Sleep state time will depend on *N_pC_*. Next, we characterize the device current consumption in the states involved in each cycle.

### 5.3. Current Consumption Profile

In this section, we present the current consumption profile of all the states involved in a fragmented SCHC Packet transfer, which have been introduced in Section 5.2. These current consumption profiles are obtained by using the experimental setup shown in Section 5.1. All individual results provided correspond to the average of 10 individual experiments. For a given scenario and set of configuration parameters, we found negligible differences among the individual results obtained.

#### 5.3.1. Sleep and Wake-Up States Current Consumption Profile

Most Sigfox devices are battery-powered. Therefore, to improve battery lifetime, they must remain in Sleep state most of the time, and only wake up for communication. The LoPy4 supports two sleep modes: the light sleep mode and the deep sleep mode. In the light sleep mode, most peripherals and CPU are clock-gated, and voltage consumption is reduced, which allows for a reduced wake-up time. In the deep sleep mode, the CPU and all peripherals are stopped, which reduces the current consumption to the minimum but increases wake-up time.

The Wake-up state current consumption and duration depends on the sleep mode used. Table 1 and Table 2 present the Wake-up state duration and current consumption, and the Sleep state current consumption, for the light sleep mode and the deep sleep mode, respectively.

**Figure 9:**
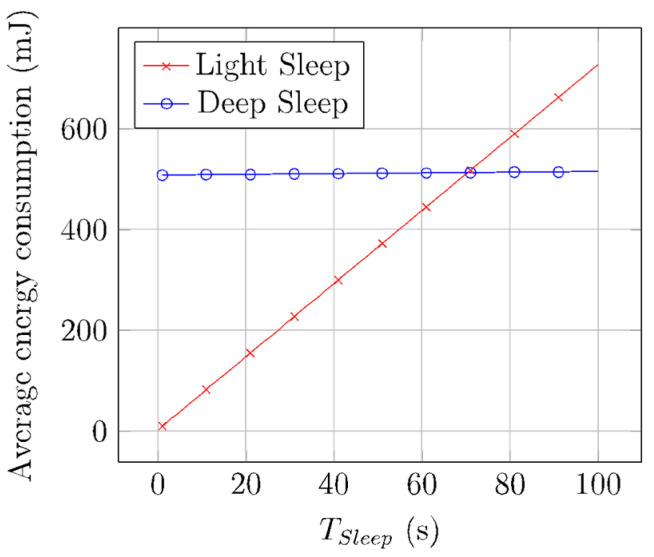
Average energy consumption of Wake-up and Sleep states for different *T_Sleep_*, values, and for light and deep sleep mode.

As shown in Table 3 and Table 4, there is a large difference between Wake-up state and Sleep state time and current consumption for light and deep sleep modes. The light sleep mode has a shorter Wake-up state time but a greater sleep current. The corresponding average energy consumption is illustrated in Figure 9. For short sleep periods, light sleep is more efficient energywise, as the Wake-up state time is shorter. For long sleep intervals, deep sleep becomes more efficient, since the longer Wake-up state duration is compensated by the ultralow deep sleep current consumption in the Sleep state.

#### 5.3.2. SCHC Fragmentation States Current Consumption Profile

Table 5 presents the SCHC fragmentation states duration and their corresponding current consumption.

**Figure 10:**
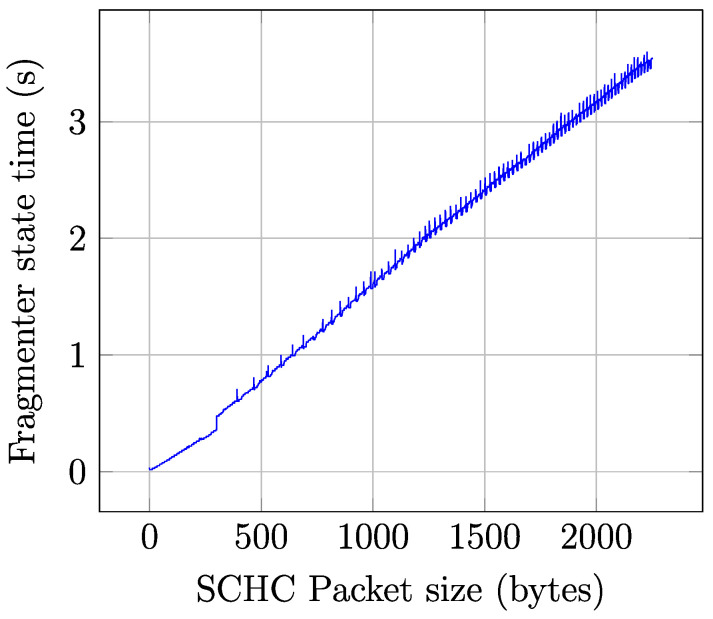
Fragmenter state time as a function of the SCHC Packet size.

In contrast with the durations of the Frag Prep, Inter Frag, and Post Frag states, which are constant, the Fragmenter state duration is proportional to the SCHC Packet size. Figure 10 illustrates the Fragmenter state duration, as a function of the SCHC Packet size, for SCHC Packet sizes between 1 and 2250 bytes. For small SCHC Packet sizes, the impact of the fragmentation process on time is negligible. However, as SCHC Packet size increases, the Fragmenter state duration becomes more significant (up to 3.54 s for a SCHC Packet size of 2250 bytes). Note that the Fragmenter state is only present once in each SCHC Packet transfer.

#### 5.3.3. U-Proc Current Consumption Profile

**Figure 11:**
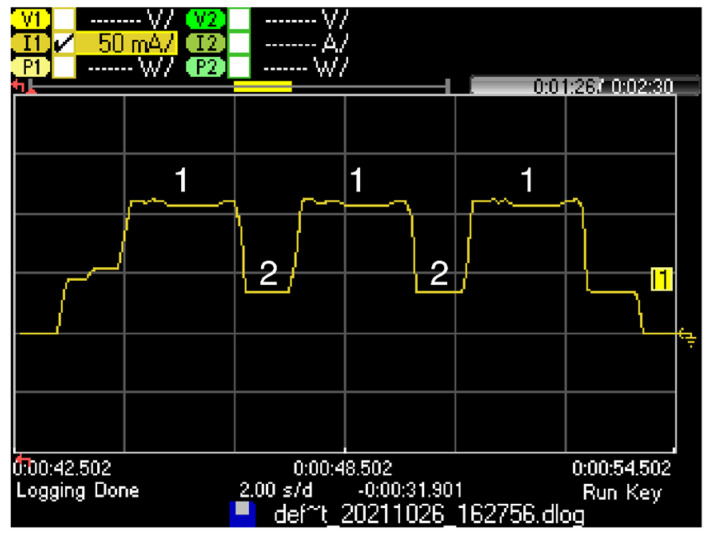
Current consumption profile of a LoPy4 device performing a U-Proc. In this measurement, the Sigfox UL frame payload size is 12 bytes, equivalent to a Regular (not All-0) SCHC Fragment carrying one tile.

In the Frag Prep state or in the Inter Frag state, the following SCHC Fragment is prepared to be transmitted by using one of the two Sigfox procedures (i.e., U-Proc or B-Proc), depending on the SCHC Fragment type (i.e., Regular (not All-0), All-0 or All-1). If the SCHC Fragment is a Regular (not All-0) SCHC Fragment, the U-Proc is selected. Figure 11 shows the U-Proc current consumption profile, as measured on the LoPy4.

The U-Proc comprises three substates: Transmission (Substate 1), Wait next transmission (Substate 2), and Cooldown (Substate 3). Table 6 presents the duration and current consumption of these substates along with their notations. Substate 1 is repeated three times, as the UL frame is sent by using three different frequencies. Substate 2 is present twice, between two consecutive transmissions. After sending the UL frame, the Sigfox radio module enters Substate 3 before transiting to the Inter Frag or Post Frag states, or before handling other processes.

The transmission current measured value, denoted *I_Tx_*, is greater than the one presented in the LoPy4 datasheet [27], as it involves the MCU in addition to the Sigfox radio module, and the input voltage is different (our experiments are performed using 3.5 V, whereas datasheet values are provided for 5 V).

Let *I_U-Proc_* denote the average current consumption of a *U-Proc*. Using the notation of Table 6, *I_U-Proc_* can be calculated as follows:

(1) $$I_{U\text{-}Proc}\;\left(\mathrm{mA}\right)=\frac{3*I_{Tx}*T_{Tx}+2*\;I_{Wait\_Tx}*T_{Wait\_Tx}+I_{Cool}*T_{Cool}}{T_{U\text{-}Proc}},$$

where *T_U-proc_* denotes the total *U-Proc* duration and can be calculated as follows:

(2) $$T_{U\text{-}Proc}\;\left(s\right)=3*T_{Tx}+2*T_{Wait_{Tx}}+T_{Cool}$$

#### 5.3.4. B-Proc Current Consumption Profile

**Figure 12:**
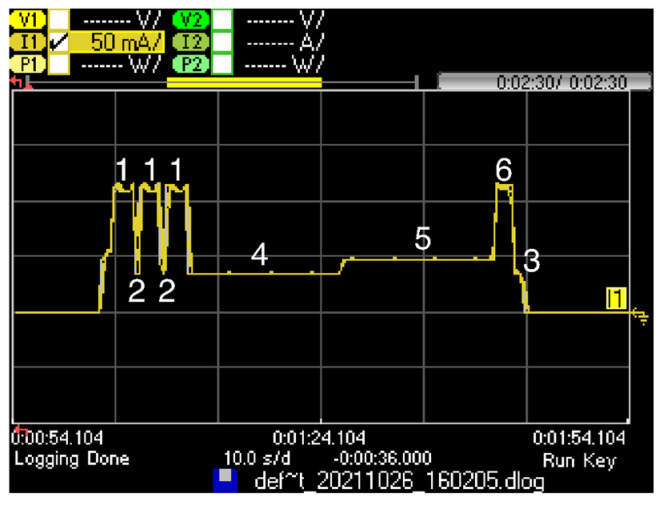
Current consumption profile of a LoPy4 in a B-Proc, with an UL frame payload of 12 bytes, equivalent to an All-0 SCHC Fragment or an All-1 SCHC Fragment carrying one tile. A DL frame is received by the device, which subsequently sends a confirmation control frame.

All-0 and All-1 SCHC Fragments need to open a DL reception window to offer the SCHC receiver the opportunity to transmit a SCHC ACK. To this end, the transmission of such fragments is performed by using a B-Proc. Figure 12 shows the B-Proc current consumption profile, as measured on the LoPy4 module. The B-Proc comprises six substates: Transmission (substate 1), Wait next transmission (substate 2), Wait for reception (substate 4), Reception (substate 5), Confirmation (substate 6), and Cooldown (substate 3). Table 7 presents the measured duration and current consumption for each substate of the B-Proc.

In a similar way to a *U-Proc*, the UL frame in a B-Proc is transmitted by using 3 different frequency channels; therefore, substate 1 is present three times, and the substate 2 is present twice, between transmissions. After the third transmission for the UL frame, the Sigfox module waits for a fixed duration time interval in substate 4 and opens the reception window in substate 5. The duration of substate 5 depends on when the DL frame is received. After receiving the DL frame, the confirmation control frame is sent in substate 6. In substate 3, the device radio cools down before allowing the MCU to perform other operations. In case the Sigfox network and/or application does not send any DL frame to the device, or the device does not receive it, substate 6 is not present, and substate 5 duration is the maximum one (i.e., *T_RX MAX_*, which is equal to 25 s in RC1).

The average current consumption of a *B-Proc* when a *DL* frame is received by the device, denoted *I_B-Proc-DL_*, can be obtained as follows:

(3) $$I_{B\text{-}Proc\text{-}DL}\;\left(\mathrm{mA}\right)=\frac{3*I_{Tx}*\;T_{Tx}+2*\;I_{Wait\_Tx}*T_{Wait\_Tx}+I_{Wait\_Rx}*T_{Wait\_Rx}+I_{Conf}*T_{Conf}+I_{Cool}*T_{Cool}}{T_{B\text{-}Proc\text{-}DL}},$$

where *T_B-Proc-DL_* can be calculated as follows:

(4) $$T_{B\text{-}Proc\text{-}DL}\;\left(s\right)=3*T_{Tx}+2*T_{Wait_{Tx}}+T_{Wait_{Rx}}+T_{Conf}+T_{Cool}.$$

The average current consumption of a B-Proc when a DL frame is not received by the device, denoted *I_B-Proc-NO-DL_*, can be obtained as follows:

(5) $$I_{B\text{-}Proc\text{-}NO\text{-}DL}\;\left(\mathrm{mA}\right)=\frac{3*I_{Tx}*\;T_{Tx}+2*\;I_{Wait\_Tx}*T_{Wait\_Tx}+I_{Wait\_Rx}*T_{Wait\_Rx}+I_{Cool}*T_{Cool}}{T_{B\text{-}Proc\text{-}NO\text{-}DL}},$$

where *T_B-Proc-NO-DL_* can be obtained as follows:

(6) $$T_{B\text{-}Proc\text{-}NO\text{-}DL}\;\left(s\right)=3*T_{Tx}+2*T_{Wait\_Tx}+T_{Wait\_Rx}+T_{Cool}$$

### 5.4. SCHC Packet Transfer Current and Energy Consumption Model

In this subsection, we model the SCHC Packet transfer current and energy consumption over Sigfox. To this end, we first calculate the number of U-Proc and B-Proc required to transfer a SCHC Packet. Then, we derive a current and energy consumption model of SCHC Packet transfer in two cases: (i) single and (ii) periodic SCHC Packet transfers.

#### 5.4.1. Number of U-Proc and B-Proc

The number of *U-Proc* (*N_U-Proc_*) required to transfer a *SCHC* Packet of size *L_SCHC_* can be obtained as follows:

(7) $$N_{U\text{-}Proc}=\lceil \frac{L_{SCHC}}{L_{UL}-L_{Header}}\rceil -\lceil \frac{L_{SCHC}}{WINDOW\_SIZE*t\;}\rceil ,$$

where *L_UL_* is the maximum Sigfox *UL* frame payload size of 12 bytes, and *L_Header_* is the size of the SCHC Fragment header.

The number of *B-Proc* with a DL frame (*N_B-Proc-DL_*) required to transfer a SCHC Packet without fragment losses is equal to 1. Under such conditions, the number of B-proc with no *DL* (*N_B-Proc-NO-DL_*) can be obtained as follows:

(8) $$N_{B\text{-}Proc\text{-}NO\text{-}DL}=\lceil \frac{L_{SCHC}}{WINDOW\_SIZE*t\;}\rceil -1,$$

#### 5.4.2. Single SCHC Packet Transfer Model

**Figure 13:**
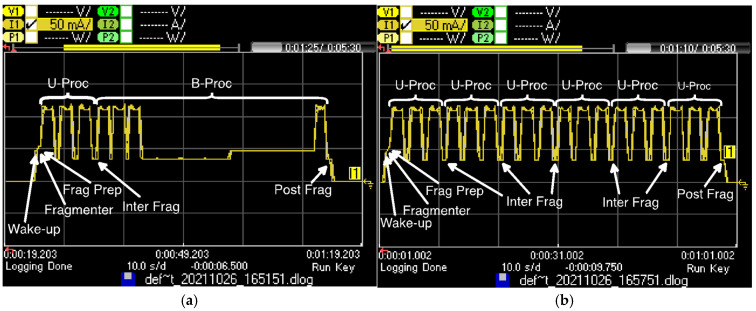
Current consumption of two SCHC Packet transfer examples: (**a**) a 22-byte SCHC Packet is sent completely and a SCHC ACK is received; (**b**) six SCHC Fragments are sent back-to-back before the device returns to the Sleep state in the first transfer cycle of a 77-byte SCHC Packet.

The number of cycles required to transfer a single *SCHC* Packet (*N_C_)* will depend on the fragment sending strategy, i.e., on the *N_pC_* value. Figure 13 illustrates the current consumption of (a) a 22-byte SCHC Packet transfer for *N_C_* = 1 and *N_pC_* = 2 and (b) the first transfer cycle of a 77-byte SCHC Packet for *N_C_* = 2 and *N_pC_* = 6. The figure shows that the number of Inter Frag states increases with *N_pC_*.

Note that *N_C_* is related to the number of Wake-up and Frag Prep and Post Frag states required to complete the SCHC Packet transfer. By sending up to 6 SCHC Fragments back-to-back (i.e., $N_{pC}\;\leq 6)$, the number of Wake-up, Frag Prep, and Post Frag states is minimized, when compared to $N_{pC}=1$.

Once the sending strategy is selected, i.e., the *N_pC_* value is chosen, *N_C_* can be calculated as follows:

(9) $$N_{C}=\lceil \frac{N_{U\text{-}Proc}+N_{B\text{-}Proc\text{-}NO\text{-}DL}+N_{B\text{-}Proc\text{-}DL}}{N_{pC}}\rceil .$$

The number of Wake-up, Frag Prep, and Post Frag states (denoted *N_Wake-up_*, *N_Prep_*, and *N_Post_*) is equal to *N_C_*, as each time the device transmits one or several back-to-back SCHC Fragments, it must wake up, prepare the next SCHC Fragment, and then do the SCHC Fragment post processing in the Post Frag state before returning to the Sleep state. We define the SCHC Packet active time (*T_act_*) as the time the device is not in the Sleep state. *T_act_* can be obtained as follows:

(10) $$\begin{array}{c} T_{act}\;\left(s\right)=T_{Frag}+N_{C}*\left(T_{wake\text{-}up}+T_{Prep}+T_{Post}+T_{Inter}*\left(N_{pC}-1\right)\right)+N_{U\text{-}Proc}*T_{U\text{-}Proc}+N_{B\text{-}Proc\text{-}NO\text{-}DL}*T_{B\text{-}Proc\text{-}NO\text{-}DL}\\ \;+T_{B\text{-}Proc\text{-}DL}. \end{array}$$

The SCHC Packet active time current consumption (*I_act_*) can be calculated as follows:

(11) $$\begin{array}{c} I_{act}\;\left(\mathrm{mA}\right)=\frac{1}{T_{act}}(I_{Frag}*T_{Frag}+N_{C}*\left(I_{wake\text{-}up}*T_{wake\text{-}up}+I_{Prep}*T_{Prep}+I_{Post}*T_{Post}+I_{Inter}*T_{Inter}*\left(N_{pC}-1\right)\right)\\ \;+N_{U\text{-}Proc}*I_{U\text{-}Proc}*T_{U\text{-}Proc}+N_{B\text{-}Proc\text{-}NO\text{-}DL}*I_{B\text{-}Proc\text{-}NO\text{-}DL}*T_{B\text{-}Proc\text{-}NO\text{-}DL}+I_{B\text{-}Proc\text{-}DL}*T_{B\text{-}Proc\text{-}DL}) \end{array}$$

As explained in Section 3.2, in RC1, a transmission procedure can only be started, at least, every 600 s, denoted *T_per Proc_*. Therefore, *T_act_* is only a small part of the total SCHC Packet transfer time (*T_SCHC_*). The latter can be obtained as follows:

(12) $$T_{SCHC}=\left(N_{U\text{-}Proc}+N_{B\text{-}Proc\text{-}DL}+N_{B\text{-}Proc\text{-}NO\text{-}DL}\right)*T_{per\;Proc},$$

Note that *T_SCHC_* is independent of *N_C_*, as the same total wait time has to be enforced, regardless of whether up to 6 messages are sent back-to-back per cycle ($N_{pC}\;\leq 6)$ or one message is sent per cycle ($N_{pC}=1)$. The differences in *N_C_* are reflected in *T_act_*. Therefore, the amount of time that the device is required to be in the Sleep state to comply with duty cycle restrictions for the transfer of a SCHC Packet, denoted *T_Sleep_*, can be calculated as follows:

(13) $$T_{Sleep}=T_{SCHC}-T_{act}.$$

Finally, the average current consumption of a *SCHC* Packet transfer over Sigfox (*I_SCHC_*) can be calculated as follows:

(14) $$I_{SCHC}=\frac{I_{act}*T_{act}+T_{Sleep}*I_{Sleep}}{T_{SCHC}}.$$

In addition, the average energy consumed in a *SCHC* Packet transfer can be determined as follows:

(15) $$E_{SCHC}=I_{SCHC}*V*T_{SCHC},$$

where *V* denotes the voltage supplied to the Sigfox device.

### 5.5. Periodic SCHC Packet Transfer Energy Performance Metrics

This subsection presents the metrics used to evaluate the energy performance of SCHC over Sigfox, for a device that transfers a SCHC Packet periodically. These metrics are (i) the average current consumption, (ii) the SCHC Packet transfer energy cost, and (iii) the device lifetime.

We assume that the device starts a SCHC Packet transfer (by sending the first fragment) every time period *T_p_*. Note that the minimum possible *T_p_* value, denoted *T_p_min_*, should be equal to *T_SCHC_*. After a SCHC Packet transfer, the device will wait in the Sleep state for *T_Wait_* until *T_p_* time has passed since the start of the previous *SCHC* Packet transfer. *T_p_* can be calculated as follows:

(16) $$T_{p}=T_{SCHC}+T_{Wait}.$$

During the wait period between SCHC Packet transfers, the device is in the Sleep state, consuming a current of *I_Sleep_*. Otherwise, the device transfers a SCHC Packet, with an average current consumption of *I_SCHC_*. In consequence, the average current consumption of periodic SCHC Packet transfers (*I_p_*) can be obtained as follows:

(17) $$I_{p}=\frac{I_{SCHC}*T_{SCHC}+I_{Sleep}*T_{Wait}}{T_{p}}.$$

The energy consumed by a device performing periodic *SCHC* Packet transfers over an interval of duration *T_p_* can be obtained as follows:

(18) $$E_{p}=I_{p}*T_{p}*V.$$

Sigfox devices are commonly battery-operated, and therefore, device lifetime calculation is crucial to the performance of *SCHC* Packet transfer over Sigfox. In order to calculate the device lifetime, the battery capacity must also be taken into consideration. Let *C_p_* denote the battery capacity (typically expressed in mAh). The device lifetime, *LT*, can be calculated as follows:

(19) $$LT=\frac{C_{p}}{I_{p}}.$$

## 6. Evaluation

In this section, we evaluate energy-related performance parameters for single and periodic SCHC Packet transfers over Sigfox. First, we present the SCHC Packet current consumption and energy cost, for light and deep sleep modes, for different sending strategies. Then, we evaluate periodic SCHC Packet transfers, in terms of current consumption, energy cost and device lifetime.

### 6.1. SCHC Packet Current and Energy Consumption

**Figure 14:**
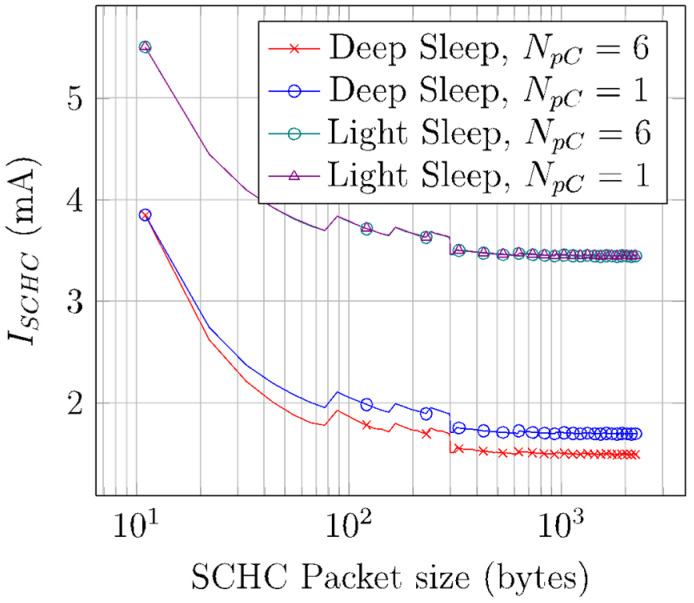
Average current consumption of a SCHC Packet transfer over Sigfox.

Figure 14 depicts *I_SCHC_* for SCHC Packet sizes between 11 and 2250 bytes, for deep sleep and light sleep, and for *N_pC_* values equal to 1 and 6. *I_SCHC_* values are obtained by using Equation (14). As SCHC Packet size increases, *T_Sleep_* increases as well due to duty cycle restrictions. In consequence, *I_SCHC_* decreases, since the device remains in sleep mode for a greater percentage of time (with a sleep current of 40 µA for deep sleep and 42 mA for light sleep).

Note that, for small SCHC Packet sizes, the sleep time versus active time ratio increases rapidly with SCHC Packet size. Such ratio is only 14 for an 11-byte SCHC Packet, while it increases to 52 for a 350-byte SCHC Packet. As the SCHC Packet size increases beyond 350 bytes, the same ratio tends asymptotically to a value of ~54. Therefore, *I_SCHC_* becomes stable between 1.36 mA and 1.32 mA, for the deep sleep mode, and equal to 3.44 mA for the light sleep mode. The *I_SCHC_* stepwise behavior that happens for small SCHC Packet sizes is due to each additional window needed to perform the SCHC Packet transfer (which increases current consumption due to the corresponding additional B-Proc). The larger step at a SCHC Packet size of 300 bytes is due to the change from a 1-byte to a 2-byte SCHC header at that value.

**Figure 15:**
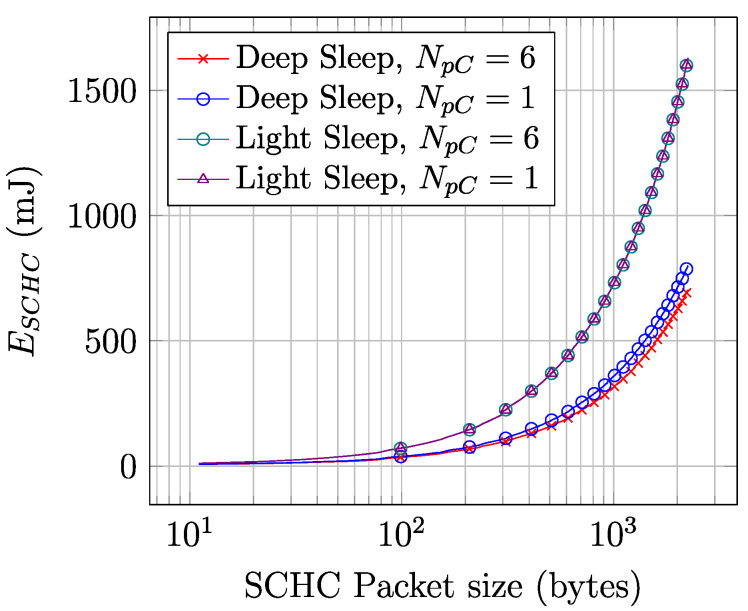
Energy consumed by a device to perform a SCHC Packet transfer over Sigfox.

Figure 15 illustrates the energy consumed by a device to perform a SCHC Packet transfer, for SCHC Packet sizes between 11 and 2250 bytes. The depicted values are obtained by using Equation (15). The energy consumption increases linearly with SCHC Packet size. Despite the fact that the average current consumption of a SCHC Packet transfer is relatively constant for SCHC Packet sizes beyond 350 bytes, the increase of SCHC Packet transfer duration with SCHC Packet size is reflected as a SCHC Packet transfer energy consumption increase.

### 6.2. Periodic SCHC Packet Transfer Energy Performance

Table 8 presents the SCHC Packet sizes used for the periodic SCHC Packet transfer energy performance evaluation, along with the corresponding values of *N_U-Proc_, N_B-Proc-NO-DL_*, and the number of windows required for a single SCHC Packet transfer. The considered SCHC Packet sizes allow to test different values for *N_U-Proc_*, *N_B-Proc-NO-DL_*, and number of windows, for single-byte and two-byte SCHC header sizes. Moreover, Table 8 also provides the *T_p_min_* value for each SCHC Packet size, and the number of SCHC Packets per day that can be transferred with a SCHC Packet sending period equal to *T_p_min_*.

**Figure 16:**
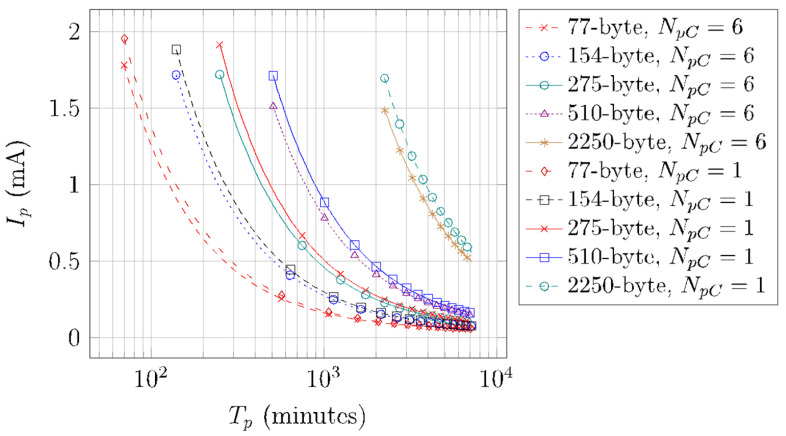
Average current consumption of a device performing periodic SCHC Packet transfers over Sigfox, for different *T_p_*, *N_pC_*, and SCHC Packet size values.

Figure 16 illustrates the average current consumption of a device that performs periodic SCHC Packet transfers, *I_p_*, for different SCHC Packet sizes, *N_pC_* values of 1 and 6. We only consider the deep sleep mode, since it is more energy-efficient than the light sleep mode. The depicted values are obtained by using Equation (17). Note that all curves do not start at the same *T_p_* value, since the minimum *T_p_* (*T_p_min_*) value is equal to *T_SCHC_* and depends on the SCHC Packet size (see Table 8). As *T_p_* increases, *I_p_* decreases for all SCHC Packet sizes, since *T_sleep_* increases, reducing the average current consumption. As shown in Figure 16, for a given SCHC Packet size, *N_pC_* = 1 consumes a greater amount of current than *N_pC_* = 6, since with the latter, the number of Wake-up, Frag Prep, and Post Frag states (and thus, their contribution to current consumption) is minimized. As the SCHC Packet size increases, the average current consumption differences between the considered *N_pC_* values increase.

**Figure 17:**
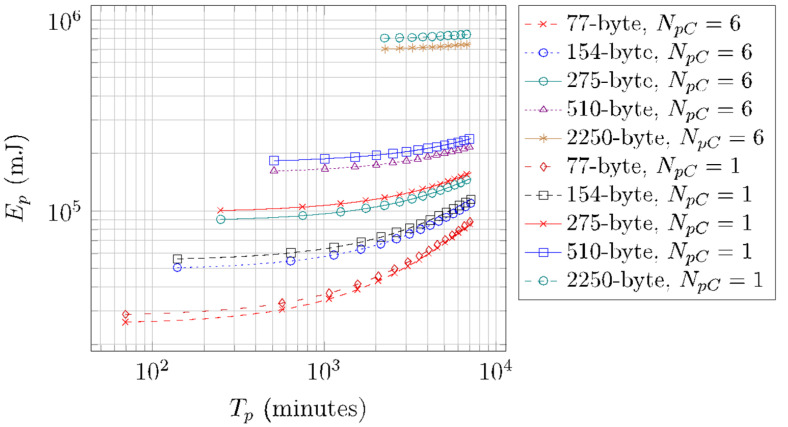
Energy consumption of a SCHC Packet transfer over a period *T_p_*, for different *T_p_*, *N_pC_*, and SCHC Packet size values.

Figure 17 illustrates the energy consumption of a SCHC Packet transfer over a period *T_p_*, for different SCHC Packet sizes, *N_pC_* values of 1 and 6, and for the deep sleep mode. The depicted values are obtained by using Equation (18). This performance parameter increases linearly with *T_p_*. This increase is small, since as *T_p_* increases, the device remains in sleep mode for a greater amount of time, which increases energy consumption, albeit to a small extent.

**Figure 18:**
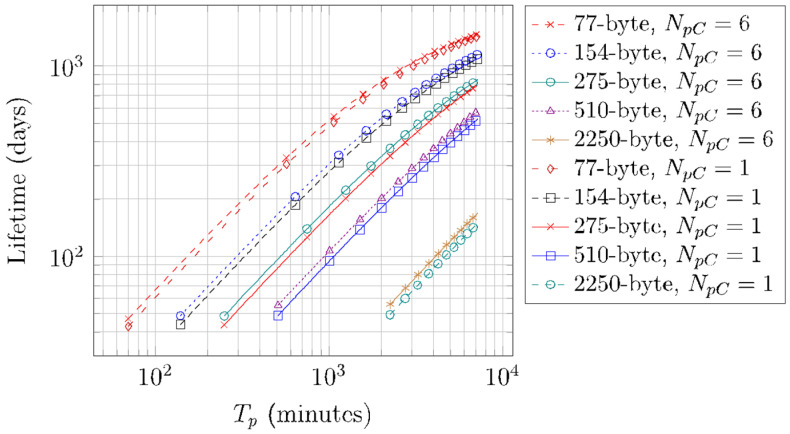
Lifetime of a battery-operated device performing periodic SCHC Packet transfers over Sigfox, for different *T_p_*, *N_pC_*, and SCHC Packet size values.

Finally, Figure 18 shows the results obtained by using Equation (19) regarding the lifetime of a device that performs periodic SCHC Packet transfers, for different SCHC Packet sizes, for *N_pC_* values of 1 and 6, and for the deep sleep mode. The battery capacity assumed in our calculation is of 2000 mAh. Recall that *T_p min_* = *T_SCHC_*. *T_p min_* ranges from 70 min for a 77-byte SCHC Packet to 2250 min for a 2250-byte SCHC Packet (see Table 8).

For the corresponding *T_p_min_* and *N_pC_* = 1, the device lifetime yields the smallest values, with a value of 42 days for a 77-byte SCHC Packet size, and 49 days for a 2250-byte SCHC Packet size. Note that there are large differences between the *T_p min_* value for specific SCHC Packet sizes, which in turn increase device lifetime, as the device spends more time in Sleep mode, and is involved in a lower number of Fragmenter states. Indeed, for a fixed *T_p_* value, as SCHC Packet size increases, more U-Proc and B-Proc are required, with the corresponding energy consumption increase and device lifetime decrease.

On the other hand, device lifetime increases asymptotically with *T_p_*. For a *T_p_* value of 5 days and for *N_pC_* = 6, and for a 77-byte SCHC Packet size, the device lifetime is 1464 days (i.e., more than 4 years). For the same *T_p_* and *N_pC_* values, and for a 2250-byte SCHC Packet size, the device lifetime is 168 days.

The device lifetime differences for *N_pC_* = 1 and *N_pC_* = 6 decrease with SCHC Packet size, due to the consequent increase of sleep time during the SCHC Packet transfer, reducing the impact of the time spent in the Wake-up, Frag Prep, and Post Frag states. For the 77-byte SCHC Packet size, such difference ranges from 4 days (*T_p_min_* = 70 min) to 42 days (*T_p_* = 5 days), whereas for the 2250-byte SCHC Packet size, the differences range from 6 days (*T_p_min_* = 2250 min) to 19 days (*T_p_* = 5 days).

## 7. Conclusions

In this paper, we have presented a model and an evaluation of the device current and energy consumption of reliable packet fragmentation by using SCHC over Sigfox. We built our model by measuring the current consumption and duration of the states involved in a SCHC Packet transfer on a real Sigfox device.

The average current consumption of a single SCHC Packet transfer decreases as the SCHC Packet size increases since the device spends more time in the Sleep state, due to the need to conform to the RC1 duty cycle restrictions. For periodic SCHC Packet transfers, the average current consumption decreases with the SCHC Packet size and with the time between transfers. In contrast, the average energy consumption increases linearly with SCHC Packet size, due to the energy consumed while the device is in the Sleep state.

We analyzed two fragment transmission strategies, which are compliant with RC1 duty cycle restrictions: sending one or up to six back-to-back SCHC Fragments per cycle, respectively. The latter is more energy-efficient. In addition, we evaluated the light and deep sleep modes provided by the Sigfox device used.

Considering a 2000 mAh battery, and with only one SCHC Fragment sent per cycle, the minimum device lifetime (which corresponds to the smallest possible packet sending period) is 49 or 42 days, for SCHC Packet sizes of 2250 bytes or 77 bytes, with a period of 2250 min or 70 min, respectively. On the other hand, if SCHC Packets are sent every 5 days, and up to 6 SCHC Fragments are sent back-to-back per cycle, the device lifetime is 168 days or 1464 days, for SCHC Packet sizes of 2250 bytes or 77 bytes, respectively. The obtained results highlight that the SCHC Packet size, the packet sending period, and the number of SCHC Fragments per cycle have a significant impact on the device lifetime.

## Figures and Tables

**Table 1:** List of references that evaluate Sigfox energy performance.

Reference	Sigfox Module	Battery Capacity (mAh)	Sending Period	Lifetime (Years)	Packet Fragmentation
[17]	Not specified	Not specified	Not specified	Not specified	No
[18]	ATA8520 Sigfox	2400	10 min	1.5 (at 600 bit/s) 2.5 (at 100 bit/s)	No
[19]	Onsemi AXSF	2400	Not specified	Not specified	No
[20]	Telit LE51-868/DIP	1000	60 min	4 (at 600 bit/s)	No
[21]	TD1202	1500 × 2	24 h	25 (at 100 bit/s)	No
[22]	TD1207R/08R	Not specified	Not specified	Not specified	No
[23]	Telit LE51-868S	1700	60 min	1.5 (at 100 bit/s)	No
[24]	AX-Sigfox	1500	10 min	1 (at 100 bit/s)	No
[25]	Not specified	Not specified	Not specified	Not specified	No
[26]	AX-SIP-SFEU	2000	10 min	0.57 (at 100 bit/s)	No

**Table 2:** List of references related to SCHC F/R performance evaluation.

Reference	Performance Parameters	Method	Energy Performance Evaluation
[8]	Overhead, throughput, goodput, end to end delay	Simulation	No
[9]	Goodput, application capacity, efficiency, header overhead	Simulation	No
[2]	Header compression, number of fragments, number of ACKs	Theoretical	No
[10]	Channel occupancy, goodput, total delay	Simulation	No
[11]	ACK message overhead, ACK bit overhead with and without L2 headers	Theoretical	No
[12]	Error rates and patterns	Simulation	No
[13]	Packet delivery ratio, goodput per ToA	Experimental	No
[14]	Network delay, SNR, power consumption	Experimental	No
[15]	Transfer time, number of uplink and downlink messages	Theoretical, Experimental	No
[16]	Channel occupancy efficiency	Theoretical, Experimental	No

**Table 3:** Wake-up and Sleep states characterization for the light sleep mode.

States	Duration Notation	Duration (ms)	Average Current Consumption Notation	Average Current Consumption (mA)	Average Energy Consumption (mJ)
Wake-up	*T_Wake-up_*	20	*I_Wake-up_*	42	2.94
Sleep	*T_Sleep_*	-	*I_Sleep_*	2.07	-

**Table 4:** Wake-up and Sleep states characterization for the deep sleep mode.

States	Duration Notation	Duration (ms)	Average Current Consumption Notation	Average Current Consumption (mA)	Average Energy Consumption (mJ)
Wake-up	*T_Wake-up_*	2770	*I_Wake-up_*	52.4	508.02
Sleep	*T_Sleep_*	-	*I_Sleep_*	0.02	-

**Table 5:** SCHC Fragmentation states.

States	Duration Notation	Duration (ms)	Average Current Consumption Notation	Average Current Consumption (mA)
Fragmenter	*T_Frag_*	see Figure 10	*I_Frag_*	55.3
Frag Prep	*T_Prep_*	23.26	*I_Prep_*	55.3
Inter Frag	*T_Inter_*	19.07	*I_Inter_*	55.3
Post Frag	*T_Post_*	28.74	*I_Post_*	55.3

**Table 6:** U-proc substates and their corresponding duration and current consumption values.

Substates	Duration Notation	Duration (ms)	Average Current Consumption Notation	Average Current Consumption (mA)
1. Transmission	*T_Tx_*	[1120, 2080]	*I_Tx_*	112.9
2. Wait next transmission	*T_Wait_Tx_*	1000	*I_Wait_Tx_*	34.02
3. Cooldown	*T_Cool_*	1000	*I_Cool_*	33.98

**Table 7:** B-Proc substates and their corresponding duration and current consumption values.

Substates	Duration Notation	Duration (ms)	Average Current Consumption Notation	Average Current Consumption (mA)
1. Transmission	*T_Tx_*	[1120, 2080]	*I_Tx_*	112.9
2. Wait next transmission	*T_Wait_Tx_*	500	*I_Wait_Tx_*	34.02
4. Wait for reception	*T_WaitRx_*	15,556	*I_Wait_Rx_*	34.14
5. Reception	*T_Rx_*	[387, 25,000] *	*I_Rx_*	45.94
6. Confirmation	*T_Conf_*	1799	*I_Conf_*	114.95
3. Cooldown	*T_Cool_*	1000	*I_Cool_*	33.98

* The value obtained in measurements and used in the evaluation is 15,550 ms.

**Table 8:** SCHC Packet sizes used in the energy performance evaluation.

SCHC Packet Size (Bytes)	*N_U-Proc_*	*N_B-Proc-NO-DL_*	*N_B-Proc-DL_*	Number of Windows	*T_p_min_* (Minutes)	SCHC Packets per Day with *T_p_min_*
77	6	0	1	1	70	20
154	12	1	1	2	140	10
275	21	3	1	4	250	5
510	49	1	1	2	510	2
2250	217	7	1	8	2250	0.64 *

* Requires more than one day for a packet transfer.
